# Novel Filter-Based Excitation Method for Pulse Compression in Ultrasonic Sensory Systems

**DOI:** 10.3390/s26010099

**Published:** 2025-12-23

**Authors:** Álvaro Cortés, María Carmen Pérez-Rubio, Álvaro Hernández

**Affiliations:** Department of Electronics, Polytechnic School, University of Alcalá, 28805 Alcalá de Henares, Madrid, Spain; alvaro.hernandez@uah.es

**Keywords:** ultrasonic sensory systems, encoding techniques, multi-carrier modulation

## Abstract

Location-based services (LBSs) and positioning systems have spread worldwide due to the emergence of Internet of Things (IoT) and other application domains that require real-time estimation of the position of a person, tag, or asset in general in order to provide users with services and apps with added value. Whereas Global Navigation Satellite Systems (GNSSs) are well-established solutions outdoors, positioning is still an open challenge indoors, where different sensory technologies may be considered for that purpose, such as radio frequency, infrared, or ultrasounds, among others. With regard to ultrasonic systems, previous works have already developed indoor positioning systems capable of achieving accuracies in the range of centimeters but limited to a few square meters of coverage and severely affected by the Doppler effect coming from moving targets, which significantly degrades the overall positioning performance. Furthermore, the actual bandwidth available in commercial transducers often constrains the ultrasonic transmission, thus reducing the position accuracy as well. In this context, this work proposes a novel excitation and processing method for an ultrasonic positioning system, which significantly improves the transmission capabilities between an emitter and a receiver. The proposal employs a superheterodyne approach, enabling simultaneous transmission and reception of signals across multiple channels. It also adapts the bandwidths and central frequencies of the transmitted signals to the specific bandwidth characteristics of available transducers, thus optimizing the system performance. Binary spread spectrum sequences are utilized within a multicarrier modulation framework to ensure robust signal transmission. The ultrasonic signals received are then processed using filter banks and matched filtering techniques to determine the Time Differences of Arrival (TDoA) for every transmission, which are subsequently used to estimate the target position. The proposal has been modeled and successfully validated using a digital twin. Furthermore, experimental tests on the prototype have also been conducted to evaluate the system’s performance in real scenarios, comparing it against classical approaches in terms of ranging distance, signal-to-noise ratio (SNR), or multipath effects. Experimental validation demonstrates that the proposed narrowband scheme reliably operates at distances up to 40 m, compared to the 34 m limit of conventional wideband approaches. Ranging errors remain below 3 cm at 40 m, whereas the wideband scheme exhibits errors exceeding 8 cm. Furthermore, simulation results show that the narrowband scheme maintains stable operation at SNR as low as −32 dB, whereas the wideband one only achieves up to −17 dB, highlighting the significant performance advantages of the proposed approach in both experimental and simulated scenarios.

## 1. Introduction

The evolution experienced over the past few decades in localization systems has been remarkable, transforming multiple aspects of our daily lives. From smartphone navigation apps [1] and autonomous driving [2] to the Internet of Things (IoT), augmented reality, healthcare monitoring, logistic or emergency response, and the ability to determine the exact position of objects, people, and devices is vital for operational efficiency, safety, and convenience. These ongoing advances in localization technology are driven by the growing demands of an increasingly interconnected and dynamic world [3].

The development of Global Navigation Satellite Systems (GNSSs), such as GPS (Global Positioning System) [4] and Galileo, has enhanced outdoor positioning capabilities, revolutionizing our ability to navigate and determine locations in open environments. However, these technologies face significant limitations indoors, where satellite signals are often obstructed or heavily attenuated by physical structures. Consequently, indoor localization technologies have evolved to address these challenges using different types of technologies, each with unique advantages and drawbacks [5,6]. Radiofrequency (RF)-based methods, such as Bluetooth [7] or Wi-Fi [8], are broadly used since smartphones are widely available today, and most buildings are Wi-Fi-enabled or can be easily provided with BLE (Bluetooth Low Energy) tags. Although effective in certain situations, these methods often deal with challenges in terms of precision, coverage, high dependence on the distribution of access points, or fluctuation of Received Signal Strength Indicators (RSSIs) [9]. Optical technologies, including image recognition and infrared (IR) light [10], offer accurate localization, but can be affected by lighting conditions, large computational load, or limitations regarding privacy issues in the case of cameras. Inertial Navigation Systems (INSs) [11] provide relative positioning with low-cost sensors; however, the noise is cumulative, so the estimated position will drift after a short time. Therefore, they are normally used in combination with other technologies [12].

Acoustic technology, which is the focus of this work, provides a low-cost solution with centimeter-level precision [13] over short distances [14,15]. This is due to the low propagation speed and high attenuation of sound through the air. It is usually preferred for the ultrasonic range to avoid audible discomfort. Distance estimation with acoustic or ultrasonic signals [16] fundamentally depends on the accurate extraction of Time-of-Arrival (ToA) [17] when transmitter-receiver synchronization is available, or Time-Difference-of-Arrival (TDoA) otherwise. In indoor positioning systems, pulse compression, and matched filtering are widely adopted to enhance detection robustness under noise and multipath conditions, by maximizing the correlation between the received signal and a predefined reference waveform. Spread spectrum sequences such as Zadoff-Chu [18], LS codes [19], and Kasami codes [20] are commonly employed due to their suitable autocorrelation and cross-correlation characteristics. Thus, the precise estimation of ToA or TDoA is critical, as it directly determines the accuracy of the subsequent trilateration phase, where spatial coordinates are estimated from the derived propagation times.

### 1.1. Related Works

In recent years, research on Acoustic Local Positioning Systems (ALPSs) has mainly focused on wideband signals and advanced coding schemes to improve the accuracy in the determination of ToAs or TDoAs and the robustness against noise and multipath propagation, as shown in [21,22,23]. In addition, different modulation and encoding techniques based on Zadoff-Chu codes [24], Kasami sequences [25], and chirp signals have been evaluated to improve positioning performance under realistic conditions [26,27]. Experimental platforms and simulation tools have also been developed to facilitate the validation and adjustment of these systems [28]. However, wideband approaches usually require a processing with a bandwidth that exceeds the one available in commercial ultrasonic transducers. This aspect often degrades the encoding properties and limits the reachable ranging distances [29], thus affecting the resulting performance, together with other factors such as the strong attenuation and high directivity of the transducers [30]. Doppler and multipath effects are also key aspects, since they deteriorate the correlation functions from most sequences [31], thus degrading the resulting performance. In contrast to this dominant trend, this work proposes a narrowband-oriented architecture that applies spread-spectrum techniques to concentrate the useful energy inside the existing bandwidth and reduce the impact of noise and interference outside it, while still maintaining high accuracy and availability comparable to other previous wideband systems.

Complementary to these developments, recent research on MIMO (Multiple-Input Multiple-Output) ultrasonic systems has shown promising results for high-resolution imaging and indoor localization, including both active and device-free approaches [32,33,34]. By exploiting multiple transmitters and receivers, MIMO architectures can generate extended virtual apertures and improve spatial diversity, thereby enhancing localization accuracy and robustness against multipath propagation. Nevertheless, such systems still face significant challenges, including the limited bandwidth of practical ultrasonic transducers, reduced signal-to-noise ratio in weak or reverberant conditions, and degradation under target motion. The narrowband excitation architecture proposed in this work can help mitigate these limitations by concentrating the transmitted energy within a reduced frequency band while preserving favorable correlation properties.

In addition to the waveform design and system architecture aspects discussed above, medium access is also a critical consideration in acoustic positioning systems. Depending on the application requirements and system topology, various multiple-access techniques, such as Time Division Multiplexing (TDM) [35], Frequency Division Multiplexing (FDM) [36], and Code Division Multiplexing (CDM) [37], have been employed to enable concurrent transmissions while minimizing interference. These strategies determine how multiple emitters share the available acoustic resources and directly influence system scalability and positioning accuracy. For instance, in [38], a CDMA-based acoustic local positioning system is proposed, demonstrating robust performance in mitigating multipath propagation effects through the use of a Matching Pursuit algorithm. On the other hand, in [36], FDM and TDM techniques are integrated to improve the positioning update rate and reduce errors in acoustic indoor localization of moving targets. In terms of modulation, schemes, such as Binary Phase Shift Keying (BPSK) or Quadrature Phase Shift Keying (QPSK), are often employed [29,39]. BPSK is appreciated for its simplicity and robustness in noisy environments, whereas QPSK allows higher data rates by encoding more bits per symbol, albeit at the cost of increased noise susceptibility. The choice of the modulation scheme affects the balance between the data rate, the reliability, and the system complexity in indoor localization systems.

### 1.2. Novelty and Contributions

In this context, this work defines a novel multi-carrier modulation method for ultrasonic-based localization systems [40] dealing with narrowband spectrum. For that purpose, the signal processing for ultrasonic transmitters and receivers is defined and modeled. The proposed modulation scheme allows them to efficiently and adaptively fit the ultrasonic transmissions into the practical bandwidth available in commercial transducers [41], rendering it highly versatile and suitable for a variety of applications. By focusing the ultrasonic signals on a narrowband range, this proposal offers significant advantages in terms of the estimation of TDoAs, improving precision, reliability, and robustness, while addressing challenges associated with interference in complex environments. The main challenges addressed by this proposal are the noise distributed across the entire spectrum in conventional modulation schemes, the lack of adaptation of the transmitted signals to the frequency response of commercial transducers, resulting in poor signal quality, and the subsequent issues in processing the frequency spectrum, particularly regarding phase detection, where the coded information to be retrieved is embedded. Summing it up, the main contributions of the proposed method are as follows:

- A novel modulation scheme consisting of multiple subcarriers and a division of the available bandwidth into several subchannels has been defined for ultrasonic systems allowing multiple channel generation.
- The proposed modulation provides a significant frequency flexibility and adaptability, which facilitates its use and integration with different commercial transmitters and receivers that typically have a narrow frequency response.
- The proposed modulation is defined together with encoding techniques for each frequency subchannel, which supports the implementation of matched filtering with high robustness against noise and over long ranges.
- The proposed modulation scheme has been successfully validated in experimental tests, providing improvements in terms of range and accuracy when compared with other previous approaches, common in the literature.

The rest of the manuscript is organized as follows: Section 2 describes the signal processing proposed for the ultrasonic transmitter; similarly, Section 3 details the signal processing associated with the ultrasonic receiver; Section 4 shows some experimental results; and, finally, conclusions are discussed in Section 5.

## 2. Transmitter Architecture

**Figure 1:**
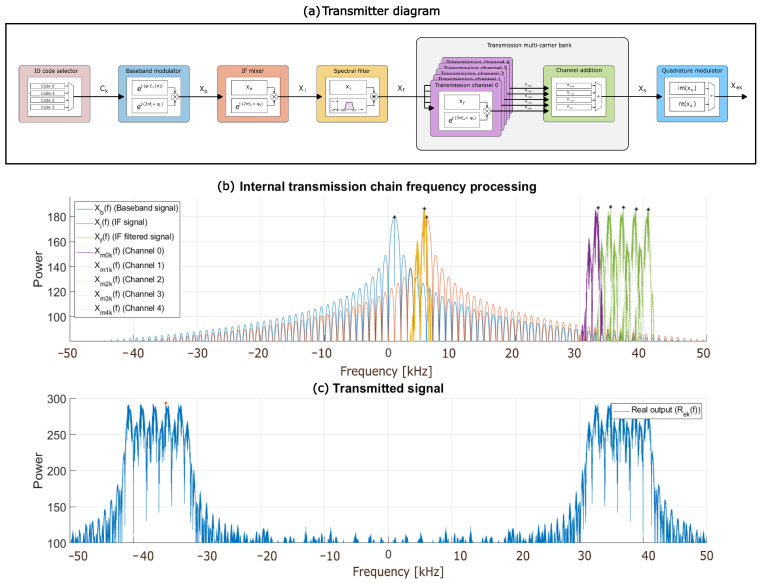
(**a**) General block diagram of the proposed transmitter. (**b**) Intermediate signals from the transmission chain in the frequency domain. (**c**) Final real signal $x_{e_{k}}\left(t\right)$ transmitted through the ultrasonic transducer.

The internal architecture of the transmitter has been designed considering the need to simultaneously use multiple ultrasonic transducers that are often involved in sensory systems, such as in positioning systems. A transmission scheme has been defined to generate the signal to be transmitted by every ultrasonic transducer *k*, 0≤k≤K−1, with K≥3 in the particular case of localization purposes. The proposed design is flexible enough to allow varying the number of transmitters or to facilitate the configuration of the system if necessary. Figure 1a shows the general block diagram of the architecture proposed for the transmitter. It starts with the selection of an identification (ID) binary code $C_{k}$ for every transmitter *k* that modulates a baseband complex carrier. The resulting baseband signal is shifted in frequency by means of an intermediate-frequency mixer, and then, it is processed by the intermediate-frequency filter, thus reducing noise and shaping the desired bandwidth. Finally, this intermediate-frequency signal is shifted again to the final transmission frequency, suitable for the ultrasonic transmission channel.

A final positioning system will require a unique identification (ID) of the signal transmitted by every ultrasonic transducer *k*. This requirement implies a module that stores the different ID codes for every transducer. These ID codes $C_{k}$ should be selected due to their suitable correlation properties, with high auto-correlation functions and low cross-correlation functions with other codes from the same family. In this proposal, 255-bit Kasami sequences have been used, which meet the above-mentioned characteristics. They make it possible to determine the TDoA for every transmitter *k* at the receiver, even if their emissions overlap in the transmission channel.

### 2.1. Baseband Modulator

The baseband modulator applies the ID binary code $C_{k}$ to modulate a complex carrier in BPSK (Binary Phase Shift Keying) at a frequency $f_{b}$. This element is designed to be highly configurable and flexible, allowing the bandwidth of the resulting modulated signal $x_{b_{k}}\left(t\right)$ for transmitter *k* to match the bandwidth available in the ultrasonic transducer. This module multiplies the carrier by a variant phasor $\phi_{C_{k}}\left(t\right)$ that depends on the bits from the code $C_{k}$ for the *k*-th emitter, as shown in (1). It is worth mentioning that the modulated signal $x_{bk}\left(t\right)$ concentrates its energy within the desired bandwidth, instead of being symmetrically distributed across the frequency spectrum. This behavior corresponds to a Single Sideband (SSB) modulation, which suppresses one of the spectral sidebands. As a result, the transmitted signal fits better the frequency response of the ultrasonic transducer, avoiding spectral replicas outside its useful bandwidth and improving the transmission efficiency.

(1) $$\begin{array}{cc} x_{b_{k}}\left(t\right) & =cos\left(2\pi \cdot f_{b}\cdot t+\phi_{C_{k}}\left(t\right)\right)+j\cdot sin\left(2\pi \cdot f_{b}\cdot t+\phi_{C_{k}}\left(t\right)\right)\\ \; & =e^{j\cdot (2\pi \cdot f_{b}\cdot t+\phi_{C_{k}}\left(t\right))} \end{array}$$

Figure 1b shows that the signal energy is placed asymmetrically in the spectrum, centered at $f_{b}=1$ kHz in this case. For clarity’s sake, the initial phase of the carrier is assumed to be zero. Although complex signal processing demands more computational resources, it plays a crucial role due to the frequency-domain behavior of the transmitted spectrum. This method helps prevent the dispersion of spectral components when combining multiple tones, eliminating the need for intermediate filtering stages in the transmitter and, thus, reducing the computational load that would be required when using real-valued signals.

### 2.2. Intermediate-Frequency Mixer

The intermediate-frequency mixer implements the frequency shifting of the input modulated signal $x_{b_{k}}\left(t\right)$, centered at $f_{b}$, to the intermediate frequency $f_{i}=6$ kHz, thus providing the signal $x_{i_{k}}\left(t\right)$, without modifying the rest of the spectral characteristics of the input signal (bandwidth, power or modulation scheme). The frequency shifting is carried out by multiplying in the time domain the input $x_{b_{k}}\left(t\right)$ with an internal complex tone at the desired frequency, in this case $f_{i_{0}}=5$ kHz. The system works in the first Nyquist window with a positive tone to increase the intermediate frequency of the signals according to (2), thus obtaining the intermediate-frequency signal $x_{i_{k}}\left(t\right)$. Note that $f_{i}=f_{b}+f_{i_{0}}=6$ kHz.

(2) $$x_{i_{k}}\left(t\right)=x_{b_{k}}\left(t\right)\cdot e^{j\cdot 2\pi \cdot f_{i_{0}}\cdot t}=e^{j\cdot (2\pi \cdot f_{b}\cdot t+\phi_{C_{k}}\left(t\right))}\cdot e^{j\cdot 2\pi \cdot f_{i_{0}}\cdot t}$$

where

(3) $$\begin{array}{cc} x_{i_{k}}\left(t\right) & =e^{j\cdot 2\pi \cdot f_{b}\cdot t+j\cdot 2\pi \cdot f_{i_{0}}\cdot t+j\cdot \phi_{C_{k}}\left(t\right)}=e^{j\cdot 2\pi \cdot \left(f_{b}+f_{i_{0}}\right)\cdot t+j\cdot \phi_{C_{k}}\left(t\right)}\\ \; & =e^{j\cdot (2\pi \cdot f_{i}\cdot t+\phi_{C_{k}}\left(t\right))} \end{array}$$

Figure 1b shows the resulting intermediate-frequency signal $x_{i_{k}}\left(t\right)$ in the frequency domain. It can be observed that the module maintains the bandwidth signal and how the central frequency is shifted from $f_{b}$ to $f_{i}$.

### 2.3. Spectral Filter

This filter and its corresponding coefficients have been selected to discard information outside the selected bandwidth. This selection allows the spectral efficiency of the system to be increased by eliminating irrelevant information and concentrating the energy to be transmitted in the desired bandwidth. The ideal response $H_{i}\left(f\right)$ of the filter is shown in (4) and (5). It is a bandpass filter centered at $f_{i}$, whose bandwidth corresponds to that of the baseband signal $x_{b_{k}}\left(t\right)$.

(4) $$H_{i}\left(f\right)=1⟶\forall \;5\;\mathrm{kHz}⩽f⩽7\;\mathrm{kHz}$$

(5) $$H_{i}\left(f\right)=0⟶\forall \;f<5\;\mathrm{kHz}\;|\;7\;\mathrm{kHz}<f$$

Assuming the ideal filter conditions, the resulting signal $x_{f_{k}}\left(t\right)$ is as follows (6):

(6) $$x_{f_{k}}\left(t\right)=h_{i}\left(t\right)*x_{i_{k}}\left(t\right)=e^{j\cdot (2\pi \cdot f_{i}\cdot t+\phi_{C_{k}}\left(t\right))}$$

As was shown in Figure 1b, the frequency-intermediate signal $x_{i_{k}}\left(t\right)$ is not bandwidth limited. This filter $H_{i}\left(f\right)$ is intended to ensure that the signal to be transmitted has as little impact as possible on the adjacent subchannels, in case of involving multiple frequency subchannels in the final signal transmission.

### 2.4. Transmission Frequency Mixer Bank

The transmission frequency mixer bank is defined as a structure of mixers that allow the incoming filtered signal $x_{f_{k}}\left(t\right)$ to be frequency-shifted to the different subchannels available in the system. In this way, the filtered signal at $f_{i}$ is shifted to a final transmission frequency subchannel *m* centered at $f_{m}$, by means of the corresponding mixer. In this work, the available ultrasonic channel may be divided into *M* subchannels that can be used to transmit different signals. At this point, without loss of generality and for clarity’s sake, the same filtered intermediate-frequency signal $x_{f_{k}}\left(t\right)$ is duplicated at every existing subchannel *m*, thus providing the system with a higher redundancy against narrowband interference. Nevertheless, note that other transmission strategies may be followed to improve the final system’s performance.

Every mixer in the bank operates with a particular frequency that allows the signal to be shifted to the correct subchannel *m*, according to (7), thus providing the resulting signal $x_{m_{k}}\left(t\right)$. Finally, the signals $x_{m_{k}}\left(t\right)$ generated by all the mixers in the bank are added, in order to obtain the complete transmission $x_{s_{k}}\left(t\right)$ of the system. Thanks to the spectral filters $H_{i}\left(f\right)$, it can be ensured that the subchannel signals $x_{m_{k}}\left(t\right)$ have a bandwidth of $\pm f_{b}$, centered at $f_{m}$. Figure 1b represents a subchannel signal $x_{m_{k}}\left(t\right)$ in the frequency domain, as well as the final added signal $x_{s_{k}}\left(t\right)$.

(7) $$\begin{array}{c} f_{m_{0}}=f_{m}-f_{i};\;f_{m}=37\;\mathrm{kHz};\\ \;f_{i}=6\;\mathrm{kHz};\;f_{m_{0}}=31\;\mathrm{kHz} \end{array}$$

(8) $$\begin{array}{cc} x_{s_{k}}\left(t\right) & =\sum_{m=0}^{M-1}x_{m_{k}}\left(t\right)=\sum_{m=0}^{M-1}e^{j(2\pi \cdot f_{i}\cdot t+\phi_{C_{k}}\left(t\right))}\cdot e^{j\cdot 2\pi \cdot f_{m_{0}}\cdot t}\\ & =\sum_{m=0}^{M-1}e^{j\cdot (2\pi \cdot f_{m}\cdot t+\phi_{C_{k}}\left(t\right))} \end{array}$$

### 2.5. Quadrature Modulator

This last modulator converts the complex signal $x_{s_{k}}\left(t\right)$ into a real signal $x_{e_{k}}\left(t\right)$ that can be transmitted through the ultrasonic transducer *k*. Note that the output signal $x_{s_{k}}\left(t\right)$ from the previous frequency mixer bank is a complex signal, with two independent components, the real one and the imaginary one (9).

(9) $$\begin{array}{cc} x_{s_{k}}\left(t\right) & =\sum_{m=0}^{M-1}e^{j\cdot (2\pi \cdot f_{m}\cdot t+\phi_{C_{k}}\left(t\right))}\\ \; & =\sum_{m=0}^{M-1}\left[cos\left(2\pi \cdot f_{m}\cdot t+\phi_{C_{k}}\left(t\right)\right)+j\cdot sin\left(2\pi \cdot f_{m}\cdot t+\phi_{C_{k}}\left(t\right)\right)\right] \end{array}$$

These two components (the cosine term and the sine term) are merged into the real output signal $x_{e_{k}}\left(t\right)$, as shown in (10), by directly adding them.

(10) $$x_{e_{k}}\left(t\right)=\sum_{m=0}^{M-1}\left[cos\left(2\pi \cdot f_{m}\cdot t+\phi_{C_{k}}\left(t\right)\right)+sin\left(2\pi \cdot f_{m}\cdot t+\phi_{C_{k}}\left(t\right)\right)\right]$$

It is worth noting that the final signal $x_{e_{k}}\left(t\right)$ to be transmitted presents spectral components on both sides of the spectrum (11).

(11) $$\begin{array}{cc} x_{e_{k}}\left(t\right) & =\sum_{m=0}^{M-1}[\frac{e^{j\cdot (2\pi \cdot f_{m}\cdot t+\phi_{C_{k}}\left(t\right))}-e^{-j\cdot (2\pi \cdot f_{m}\cdot t+\phi_{C_{k}}\left(t\right))}}{2}\\ \; & +\frac{e^{j\cdot (2\pi \cdot f_{m}\cdot t+\phi_{C_{k}}\left(t\right))}+e^{-j\cdot (2\pi \cdot f_{m}\cdot t+\phi_{C_{k}}\left(t\right))}}{2}] \end{array}$$

## 3. Receiver Architecture

**Figure 2:**
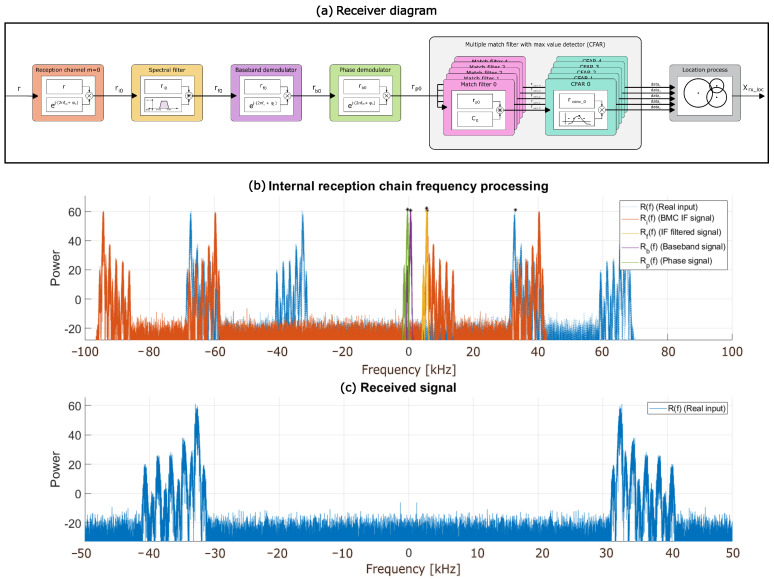
(**a**) General block diagram proposed for the ultrasonic receiver. (**b**) Intermediate signals from the reception stage in the frequency domain. (**c**) Real input signal R(f), received through the ultrasonic transducer.

The reception system performs the opposite operations and transformations to those carried out by the emitter. The aim is to improve the TDoA determination, since TDoAs are a key factor in the accuracy of the range estimates obtained afterwards. Figure 2a shows the receiver’s block diagram, starting from the signal reception. The incoming signal r(t) is shifted in frequency to an intermediate frequency, then a pass-band filter is included to discard the undesired frequency components; later, a mixer shifts the signal to the initial baseband frequency. Finally, through a non-coherent demodulation and correlation process, it is possible to detect the maximum values (peaks) in the resulting correlation functions that determine the TDoAs for the transmission from each emitter *k*. This demodulation also enables the computation of the received signal energy from both the in-phase and quadrature components, allowing the peak detection to be carried out independently of the phase ϕ, based on (12). The proposed reception system is intended to outperform classic correlation methods by allowing higher accuracy in the TDoA estimates, even in noisy environments.

(12) $$sin^{2}\left(\phi \right)+cos^{2}\left(\phi \right)=1$$

All the emitted signals $x_{e_{k}}\left(t\right)$, 0≤k≤K−1, from every transducer *k*, together with the channel noise η(t) and different replicas from multipath, will reach the receiver as indicated in (13), where $h_{T}\left(t\right)$ represents the transducer impulse response, *J* is the number of replicas for each emitted signal $x_{e_{k}}\left(t\right)$, $A_{k,j}$ is the attenuation factor, and $t_{k,j}$ the propagation delay for each replica *j*.

(13) $$r\left(t\right)=\sum_{k=0}^{K-1}\sum_{j=0}^{J-1}A_{k,j}\cdot h_{T}\left(t\right)*x_{e_{k}}\left(t-t_{k,j}\right)+\eta \left(t\right)$$

Nonetheless, for clarity’s sake, an ideal channel without multipath or noise has been considered hereinafter, so Equation (13) can be simplified into (14) for j=0.

(14) $$\begin{array}{cc} r(t) & =\sum_{k=0}^{K-1}A_{k,0}\cdot h_{T}\left(t\right)*x_{e_{k}}\left(t-t_{k,0}\right)\\ \; & =\sum_{k=0}^{K-1}A_{T_{k}}\sum_{m=0}^{M-1}\left[\frac{e^{j\cdot 2\pi \cdot f_{m}\cdot t+\phi_{C_{k}}\left(t\right)+\phi_{d_{k}}\left(t\right)}-e^{-j\cdot 2\pi \cdot f_{m}\cdot t+\phi_{C_{k}}\left(t\right)+\phi_{d_{k}}\left(t\right)}}{2}\right.\\ \; & +\left.\frac{e^{j\cdot 2\pi \cdot f_{m}\cdot t+\phi_{C_{k}}\left(t\right)+\phi_{d_{k}}\left(t\right)}+e^{-j\cdot 2\pi \cdot f_{m}\cdot t+\phi_{C_{k}}\left(t\right)+\phi_{d_{k}}\left(t\right)}}{2}\right] \end{array}$$

where $t_{k}=t_{k,0}$ is the propagation delay associated to the direct path j=0 from each emitter *k* to the receiver; $A_{T_{k}}$ includes both the effect of the channel attenuation $A_{k,0}$ and the impulse response $h_{T}\left(t\right)$ from the transmitter and the receiver; and $\phi_{d_{k}}\left(t\right)$ represents the phase shift for every subchannel *m* associated to the distance for an emitter *k*. Equation (15) simplifies the received phase information $\phi_{rk}\left(t\right)$, which combines the phase of the transmitted signal and the phase shift produced by the distance variation in the channel $\phi_{C_{k}}\left(t\right)$. Figure 2c shows an example of the spectral components of the received signal r(t), focused on *M* different subchannels, after passing through the ultrasonic transmission channel.

(15) $$\phi_{rk}\left(t\right)=\phi_{C_{k}}\left(t\right)+\phi_{d_{k}}\left(t\right)$$

### 3.1. Frequency Channel Mixer Selector

In the last stages of the transmission process, the signal $x_{f_{k}}\left(t\right)$ at the intermediate frequency $f_{i}$ was replicated at *M* different subchannels and then allocated in the frequency band of the transducer. The reverse process is carried out at the reception stage, so the frequency channel mixer selector allows the selection of the subchannel *m* to be processed. This selection is performed by a frequency shifting from the selected subchannel $f_{m}$ to the intermediate frequency $f_{i}$, as shown in (16). The resulting complex signal $r_{im}\left(t\right)$ is defined in (17).

(16) $$f_{m0}=-\left(f_{m}-f_{i}\right)$$

(17) $$\begin{array}{cc} \; & r_{im}\left(t\right)=r\left(t\right)*e^{j\cdot 2\pi \cdot f_{m0}\cdot t+\phi_{m0}}\\ \; & =\sum_{k=0}^{K-1}A_{T_{k}}\sum_{m=0}^{M-1}\left[\frac{e^{j\cdot 2\pi \cdot \left(f_{m}+f_{m0}\right)\cdot t+\phi_{rk}\left(t\right)+\phi_{m0}}-e^{-j\cdot 2\pi \cdot \left(f_{m}+f_{m0}\right)\cdot t+\phi_{rk}\left(t\right)+\phi_{m0}}}{2}\right.\\ \; & +\left.\frac{e^{j\cdot 2\pi \cdot \left(f_{m}+f_{m0}\right)\cdot t+\phi_{rk}\left(t\right)+\phi_{m0}}+e^{-j\cdot 2\pi \cdot \left(f_{m}+f_{m0}\right)\cdot t+\phi_{rk}\left(t\right)+\phi_{m0}}}{2}\right] \end{array}$$

Note that $\phi_{m0}$ is a constant value, becoming null if a phase synchronization process is implemented at the receiver. Figure 2b shows in blue the received signal r(t) and the resulting $r_{im}\left(t\right)$ in orange, both in the frequency domain. It is also possible to observe the frequency spectrum unbalance derived from the frequency channel mixer selector forced by this module, in order to center the target subchannel *m* at the intermediate frequency $f_{i}$ of the system.

### 3.2. Spectral Filter

The signal $r_{im}\left(t\right)$ is composed of multiple subchannels and needs to be cleaned before being processed. For this reason, the information received from the adjacent subchannels should be filtered out. The frequency response $H_{i}\left(f\right)$ of the filter is equal to the one defined for the transmitter, as shown in (18), extracting only the selected subchannel *m* centered at the frequency $f_{i}=6$ kHz.

(18) $$\begin{array}{c} H_{i}\left(f\right)=1⟶\forall \;5\;\mathrm{kHz}⩽f⩽7\;\mathrm{kHz}\\ H_{i}\left(f\right)=0⟶\forall \;f<5\;\mathrm{kHz}\;|\;7\;\mathrm{kHz}<f \end{array}$$

The received signal is processed in phase and quadrature. For this reason, a complex filter has been designed, which allows only the sideband to pass through, where the intermediate frequency channel of the system is centered. This filter allows all noise external to the subchannel *m* of interest is to be discarded. After the filtering, the resulting signal $r_{fm}\left(t\right)$ is shown in (19) and plotted in Figure 2b, where it is possible to observe in yellow how a subchannel *m* is centered at the intermediate frequency $f_{i}$ and isolated from the others to avoid interference.

(19) $$\begin{array}{cc} r_{fm}\left(t\right) & =r_{im}\left(t\right)*h_{i}\left(t\right)=\sum_{k=0}^{K-1}A_{T_{k}}\cdot e^{j\cdot (2\pi \cdot f_{i}\cdot t+\phi_{rk}\left(t\right)+\phi_{m0})} \end{array}$$

### 3.3. Baseband Mixer

The baseband mixer is applied to obtain in the receiving system the sine-modulated signal, generated as the main signal in the transmitting system. This block allows the central frequency of the output signal $r_{fm}\left(t\right)$ from the spectral filter to be shifted from the intermediate frequency $f_{i}$ to the baseband frequency $f_{b}$, as depicted in (21). To perform the operation, the block must generate the frequency $f_{i0}$ (20).

(20) $$f_{i0}=-\left(f_{i}-f_{b}\right)$$

(21) $$\begin{array}{cc} r_{bm}\left(t\right) & =r_{fm}\left(t\right)\cdot e^{j\cdot (2\pi \cdot f_{i0}\cdot t+\phi_{b0})}\\ \; & =\sum_{k=0}^{K-1}A_{T_{k}}\cdot \left(\sum_{m=0}^{M-1}e^{j\cdot (2\pi \cdot \left(f_{i}+f_{i_{0}}\right)\cdot t+\phi_{rk}\left(t\right)+\phi_{m0}+\phi_{b0})}\right)\\ \; & =\sum_{k=0}^{K-1}A_{T_{k}}\cdot e^{j\cdot (2\pi \cdot f_{b}\cdot t+\phi_{rk}\left(t\right)+\phi_{m0}+\phi_{b0})} \end{array}$$

Figure 2b shows the frequency spectrum of the baseband signal $r_{bm}\left(t\right)$ in purple. It represents the mixture of the baseband BPSK-modulated signals from the transmitting system, delayed with respect to each other through the communication channel.

### 3.4. Phase Demodulator

Finally, the complex subchannel baseband signal $r_{bm}\left(t\right)$ is the combination of all the BPSK baseband-transmitted signals mixed in the communication channel that arrive at the receiver. This signal has all the information required to start the determination of ranges. The phase demodulator allows us to improve this estimation by reducing the system resources: it performs a zero-span demodulation, shifting the central frequency of $r_{bm}\left(t\right)$ to 0 Hz, while maintaining the phase component in the resulting signal $r_{pm}\left(t\right)$ (22).

(22) $$\begin{array}{cc} r_{pm}\left(t\right) & =\sum_{k=0}^{K-1}A_{T_{k}}\cdot e^{j\cdot (2\pi \cdot f_{b}\cdot t+\phi_{rk}\left(t\right)+\phi_{m0}+\phi_{b0})}\cdot e^{-j\cdot (2\pi \cdot f_{b}+\phi_{p0})}\\ \; & =\sum_{k=0}^{K-1}A_{T_{k}}\cdot e^{j\cdot (\phi_{rk}\left(t\right)+\phi_{m0}+\phi_{b0}+\phi_{p0})}=\sum_{k=0}^{K-1}A_{T_{k}}\cdot e^{j\cdot \phi_{r}\left(t\right)} \end{array}$$

In (22), the signal $r_{pm}\left(t\right)$ exhibits a dependency on the variation in the received signal’s phase. This phase $\phi_{r}\left(t\right)$ encompasses the information received from all the subchannels. As can be observed in (23), it has dependencies on a constant phase over time $\phi_{cte}$, which is the initial phase of the system’s mixers, and on two phases that vary over time. One time-varying phase $\phi_{C_{k}}\left(t\right)$ corresponds to the mixing of the transmitted identification codes $C_{k}$, whereas the other $\phi_{d_{m}}\left(t\right)$ depends on the system’s spatial location.

(23) $$\phi_{r}\left(t\right)=\phi_{rk}\left(t\right)+\phi_{m0}+\phi_{b0}+\phi_{p0}=\phi_{C_{k}}\left(t\right)+\phi_{d_{m}}\left(t\right)+\phi_{cte}$$

This method significantly reduces computational complexity by reusing a sine signal, thus avoiding the computation of the arctangent of the division of the imaginary component of the signal over the real component. It also improves the correlation between the received baseband sine signal and the expected sine signals, thus providing better auto-correlation peaks and lower cross-correlation values.

### 3.5. Adaptive Filter Bank

The adaptive filter bank generates the correlation signals, where the TDoAs for the signals received from the *K* transmitters are estimated. These filters are matched to the patterns to be identified, generating a maximum value at the instant when the reception of the signal is completed. There is a filter for every code $C_{k}$ to be identified. These maximum correlation values allow us to determine the ranging distances or ToAs, in case of synchronization between the emitters and the receiver, or the TDoAs, when there is no synchronization.

It is worth noting that the signal correlation depends on the reception phase and the initial phase of the system’s mixers. In this context, a non-coherent correlation is proposed in (24), based on the quadratic sum of the in-phase signal *ℜ* and the quadrature one *ℑ*. This process ensures the maximum detection value independently of the phase $\phi_{cte}$ (12), thereby eliminating this constant component and the need to synchronize the transmitting and receiving elements.

(24) $$\begin{array}{cc} r_{am}\left(t\right) & =\sum_{k=0}^{K-1}(ℜ\left[A_{T_{k}}\cdot e^{j\cdot \phi_{r}\left(t\right)}\right]{)}^{2}+(ℑ\left[A_{T_{k}}\cdot e^{j\cdot \phi_{r}\left(t\right)}\right]{)}^{2} \end{array}$$

To clarify the mathematical foundation of the proposed non-coherent correlation and address its independence from the initial phase, we consider the received signal from each *k*-th transmitter as $A_{T_{k}}e^{j(\phi_{r}\left(t\right)+\phi_{cte})}$. The non-coherent correlation in (24) can then be expressed for the sum over all transmitters:

(25) $$\begin{array}{cc} r_{am}\left(t\right) & =\sum_{k=0}^{K-1}\left({\left[ℜ\left(A_{T_{k}}e^{j(\phi_{r}\left(t\right)+\phi_{cte})}\right)\right]}^{2}+{\left[ℑ\left(A_{T_{k}}e^{j(\phi_{r}\left(t\right)+\phi_{cte})}\right)\right]}^{2}\right)\\ \; & =\sum_{k=0}^{K-1}A_{T_{k}}^{2}\left[\cos^{2}\left(\phi_{r}\left(t\right)+\phi_{cte}\right)+\sin^{2}\left(\phi_{r}\left(t\right)+\phi_{cte}\right)\right]\\ \; & =\sum_{k=0}^{K-1}A_{T_{k}}^{2} \end{array}$$

Hence, the signal $r_{am}\left(t\right)$ depends only on the signal amplitudes $A_{T_{k}}$ and is unaffected by the constant phase term $\phi_{cte}$. This confirms that the proposed non-coherent correlation eliminates the influence of phase offsets, ensuring reliable detection and TDoA estimation even under asynchronous conditions.

At this point, the positioning only depends on the received codes $C_{k}$ and the phase variation in the received signal over time due to the range distance. In (26), the resulting correlation signal $r_{ck}\left(t\right)$ is described, where $h_{rk}$ is the impulse response of the matched filter for the code $C_{k}$.

(26) $$r_{ck}\left(t\right)=h_{rk}\left(t\right)*r_{am}\left(t\right)$$

The maximum value of the correlation signal between the processed received signal and the expected signal allows the determination of the TDoAs at the system. The minimum time interval between two correlation peaks depends on the length of the expected code, which defines the time window for TDoA detection. This enables distinguishing the direct-path signal arrival from possible replicas caused by multipath propagation. The TDoAs determined by the correlation peaks detected for the different codes $C_{k}$ allow the implementation of a positioning algorithm.

### 3.6. Evaluation of the Correlation Functions

**Figure 3:**
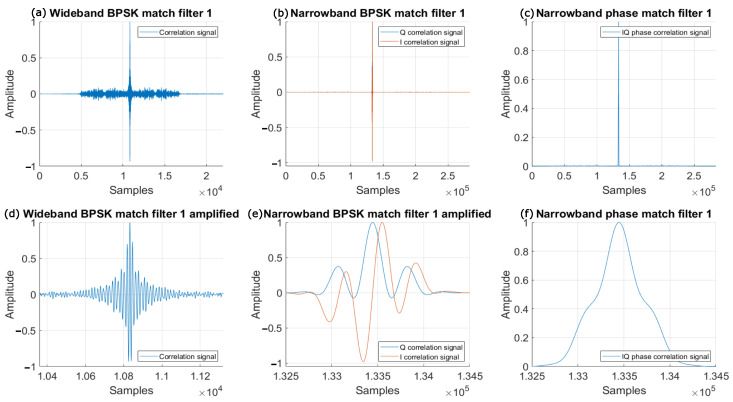
Comparison of the correlation signals obtained for: the wideband BPSK modulation (**a**) and zoom (**d**); the narrowband BPSK modulation (**b**) and zoom (**e**); and the proposed phase correlation in a narrowband BPSK modulation (**c**) and zoom (**f**).

A first aspect to consider is how the novel approach proposed here modifies the correlation properties in the matched filtering. In this way, Figure 3 shows the correlation signals for the wideband BPSK scheme defined in previous works, the narrowband BPSK scheme, and the narrowband phase correlation scheme described in this work. These signals are processed in the same way for a fair comparison, with 255-bit Kasami codes and a BPSK modulation scheme. It is possible to observe how the narrowband method proposed here presents better auto-correlation properties, with lower SMR (Sidelobe to Main lobe Ratio) values as indicated in (27), where $T_{sym}$ represents the symbol period. In the wideband case, the carrier frequency $f_{c}$ involved in the BPSK modulation is fixed at 41.67 kHz, so the symbol period is $T_{sym}=1/f_{c}=24$ μs. In the narrowband case, the symbol period is defined by the baseband carrier frequency $f_{b}$, according to $T_{sym}=1/f_{b}$ = 1 ms. The SMR metric compares the signal energy of the main correlation peak, located within the $T_{sym}$ interval, with the strongest peak found outside that range. These other peaks are usually caused by reflections of the signal (multipath) or by imperfections in the correlation functions of the selected codes. In (27) the numerator, $max\left(r_{ck}\left(t\right)\right)\{\forall t\notin \left[-T_{sym}/2,T_{sym}/2\right]$ represents the maximum value of the correlation function $r_{ck}\left(t\right)$ outside the window defined around the main lobe with a width equal to the modulation symbol $T_{sym}$. On the other hand, the denominator in (27), $max\left(r_{ck}\left(t\right)\right);\forall \left\{r_{ck}\left(t\right)\right\}$, represents that maximum correlation value, corresponding to the main lobe. In encoded ultrasonic positioning systems, this parameter serves as a standard indicator of how easily the correlation peak can be identified [42].

(27) $$SMR=\frac{\mathrm{Maximum}\;\mathrm{correlation}\;(\mathrm{side}\;\mathrm{lobe})}{\mathrm{Maximum}\;\mathrm{correlation}\;(\mathrm{main}\;\mathrm{lobe})}=\frac{max\left(r_{ck}\left(t\right)\right)\left\{\forall t\notin \left[-T_{sym}/2,T_{sym}/2\right]\right\}}{max\left(r_{ck}\left(t\right)\right);\forall \left\{r_{ck}\left(t\right)\right\}};$$

Figure 3 displays six graphs: the upper left one (a) shows the resulting correlation for a complete reception in the wideband BPSK modulation scheme; the upper middle graph (b) plots the correlation functions obtained in the narrowband BPSK modulation for both in-phase and quadrature components; and the upper right graph (c) shows the function obtained by the narrowband phase correlation proposed here. Furthermore, the lower graphs provide a close-up of the correlation’s most critical part, highlighting the maximum signal value: bottom left (d) for the wideband BPSK modulation; bottom middle (e) for the narrowband BPSK scheme; and bottom right (f) for the narrowband phase correlation. It is worth mentioning that, in the wideband modulation, the signal transmission is shorter due to its higher bandwidth, but the correlation function appears more degraded. Conversely, the narrowband approach adapts better to the transducer’s bandwidth, resulting in a more suitable correlation function that makes the detection of the maximum value easier, with lower SMR ratios.

The proposed phase correlation significantly improves the appearance of the correlation functions, allowing the result to be independent of the initial phase of the received signal. As shown in Figure 3e, the in-phase correlation signal is small when the quadrature correlation one is high, and vice versa. This variation depends on the position of the receiver with respect to the transmitter. Therefore, the quadratic sum of the in-phase and quadrature correlations is performed to obtain a final correlation signal with a constant maximum value, regardless of spatial location.

## 4. Experimental Results

Simulations and experimental tests have been conducted to evaluate the performance of the proposed narrowband methodology against the conventional wideband approach. These tests were performed in simulated environments with realistic conditions allowed by a digital twin. These conditions included several levels of ambient noise, different distances between transmitters and receivers, and various transducer bandwidths. Furthermore, experimental tests in a real scenario have been carried out to prove the system’s performance as well. The 255-bit-length Kasami codes have been considered for the transmissions, as they provide suitable correlation properties in ultrasonic systems. Nevertheless, note that other sequences, such as Zadoff-Chu codes, may also be involved. In [43], a detailed study of different sequences applied to the ultrasonic signal encoding can be found.

The ultrasonic transmitter unit involved in these tests is composed of five Prowave 328ST160 ultrasonic transducers [44] (Pro-Wave Electronic Corp., New Taipei City, Taiwan), arranged according to [45]. These transducers present an approximate bandwidth of 8 kHz. The transmitter response has been experimentally characterized in an anechoic chamber, where the transmitter was excited by a dual-channel Tabor 5062 Arbitrary Waveform Generator (Tabor Electronics Ltd., Nesher, Israel) followed by a Tabor 9200 voltage amplifier (Tabor Electronics Ltd., Nesher, Israel), ensuring a 1 ppm clock accuracy and stability [46]. As for the receiver, a Brüel & Kjær 4939 high-bandwidth omnidirectional microphone [47] (Hottinger Brüel & Kjaer GmbH, Darmstadt, Germany) with a large bandwidth of 100 kHz has been used; this microphone is supplied by the manufacturer with a calibration chart that ensures an open-circuit sensitivity of 4.3 mV/Pa with an uncertainty of 0.2 dB (95% confidence level). An Avisoft-116Hm unit (Avisoft Bioacoustics e.K., Glienicke/Nordbahn, Germany) is also involved, which amplifies the reception and provides the acquired samples to a PC through a USB link. Note that no synchronization has been implemented between the emitter and the receiver. In this context, some design parameters have been considered hereinafter: a sampling frequency of 500 kHz for the transmitter and of 100 kHz for the receiver, both meeting the Nyquist criterion and defined by the hardware equipment involved. In the wideband BPSK modulation, the carrier frequency was set at $f_{c}=41.67$ kHz. In the narrowband approach, the carrier frequency for the baseband modulator was fixed at $f_{b}=1$ kHz. Furthermore, as for the time synchronization, it is worth noting that, in the worst-case scenario, common accuracies in the range of ppm for clocks of tens of MHz might only imply an error lower than one sample in the determination of the position of the correlation peaks on the time axis, and the corresponding TDoAs. This is due to the fact that the sampling frequencies involved in the transmission and reception are 500 kHz and 100 kHz, respectively, whereas the proposed narrowband transmissions are 255 ms long, so the accumulated clock drift is not significant, taking into account that the transmitter and the receiver are not synchronized (asynchronous detection).

It is also worth mentioning that both simulations and experimental tests described hereinafter have involved the use of M=5 subchannels at the transmission in the narrowband approach. The same Kasami sequence was inserted in the five subchannels for clarity’s sake, in order to evaluate possible degradations when using several adjacent subchannels. Note that other sequences or data could be transmitted through each subchannel when necessary. On the other hand, in the receiver, only the subchannel *m* at $f_{m0}=31$ kHz was processed for evaluation. For comparison’s sake, the transmitted energy was normalized for both modulation approaches, wideband and narrowband.

### 4.1. Simulated Tests and Validation Metrics

To assess the performance achieved by the proposed narrowband method, the SMR was used as the primary performance indicator. This metric relates the amplitude of the main correlation lobe to the secondary lobe, thus determining when a correlation peak may be clearly identified or whether it presents any ambiguity. Additionally, the variation in the determination of the position of the correlation peak over time has been analyzed for both modulations, wideband and narrowband. It is important to remark that, by default, in the simulated scenarios the distance between the emitter and the receiver was fixed at 2.5 m, the signal-to-noise ratio (SNR) at 10 dB, and the Gaussian noise level at −3 dBm. Nevertheless, note that these parameters will vary in the following tests to assess their influence on the final performance. The system was configured to work without multipath, using a single emitter and a receiver with M=5 modulation subchannels.

**Figure 4:**
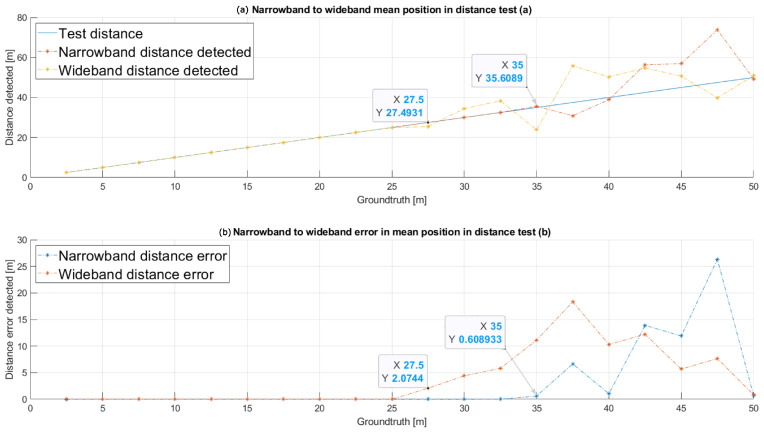
Mean distance (**a**) estimated for both the narrowband modulation and the wideband one for a range from 2.5 m to 50 m, with an SNR of 10 dB; and absolute mean error (**b**) in the distance estimation.

In the first scenario, 200 tests were conducted at 20 different distances between the emitter and the receiver, thus implying ten trials per considered distance, without varying the default values from the other aforementioned parameters. The distance between the transmitter and the receiver has increased from 2.5 m to 50 m in steps of 2.5 m. In Figure 4a, the mean distance estimates are shown for both the wideband modulation and the narrowband one. The ground truth is plotted in blue, whereas the narrowband modulation is in red and the wideband one is in yellow. Furthermore, in Figure 4b, it is possible to observe that the narrowband one presents a higher robustness in the distance determination, with lower mean absolute errors. Particularly, the narrowband approach behaves reliably up to 40 m, whereas the wideband one degrades from 25 m on.

**Figure 5:**
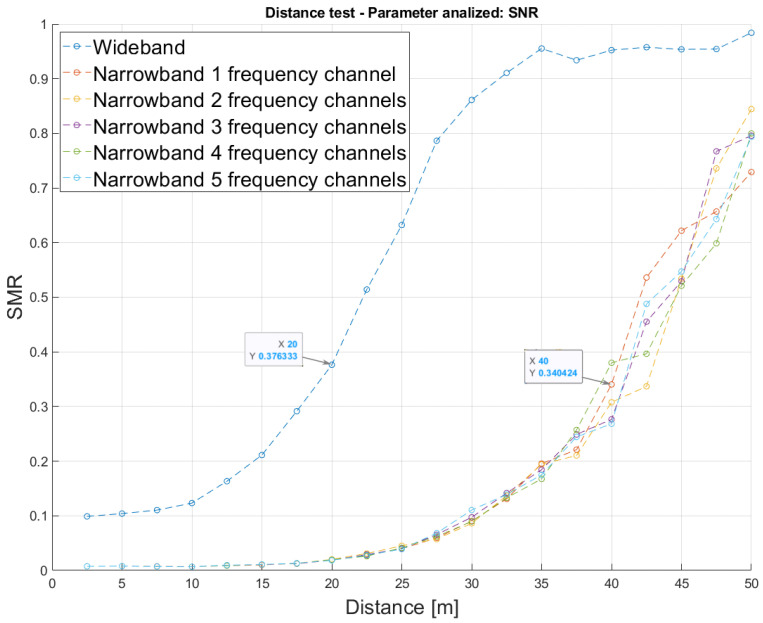
Average SMR according to the distance between the emitter and the receiver for both the narrowband and the wideband modulations, with an SNR of 10 dB and an increasing number M={1,2,3,4,5} of subchannels in the narrowband approach.

Similarly, Figure 5 shows the average SMR values as a function of the distance between the emitter and the receiver. As before, 200 simulations were run, where ten trials were carried out for every considered distance (from 2.5 m to 50 m in steps of 2.5 m). Assuming that SMR values below 50% allow a precise distance estimation, the SMR of the wideband approach increases more rapidly than that from the narrowband one as the distance increases, thus implying that the wideband modulation degrades at shorter distances than the narrowband. Furthermore, the narrowband approach has been analyzed for a varying number M={1,2,3,4,5} of subchannels involved in the modulation scheme, showing that the performance is quite similar in all these cases, thus proving the capability of the proposal to avoid interference between subchannels with an increasing number of them. In that way, from the SMR point of view, and considering the SMR limited at 50%, the wideband approach behaves suitably up to a distance of 20 m, whereas the narrowband one reaches 32.5 m.

**Figure 6:**
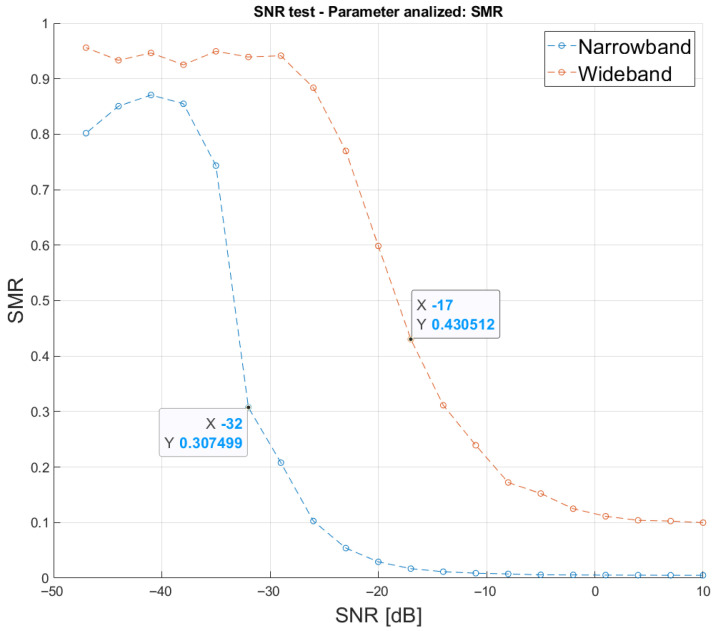
Average SMR according to the SNR for both approaches, narrowband and wideband, for a distance between the emitter and the receiver of 2.5 m and an SNR ranging from −47 dB to 10 dB in steps of 3 dB.

Another key factor when detecting the maximum value of the correlation function is the SNR of the received signal. The influence of this parameter has been studied by modifying the SNR in the simulation of the communication channel. Figure 6 presents this study conducted with SNR values ranging from −47 dB to 10 dB in steps of 3 dB, for a fixed distance between the emitter and the receiver of 2.5 m. Note that ten trials were run for every SNR value. Thanks to the Kasami sequences used to encode the ultrasonic transmissions, the signal is detectable at relatively low SNR values. The narrowband architecture, by better adapting the transmission to the transducer’s frequency response, reduces the loss of information on the transmitted signal. This enhancement allows the narrowband architecture to detect the correlation peak at lower SNRs, compared to the wideband one. Particularly, assuming an SMR limit at 50% again, the narrowband one works suitably up to an SNR of −32 dB, whereas the wideband one only reaches an SNR of −17 dB.

**Figure 7:**
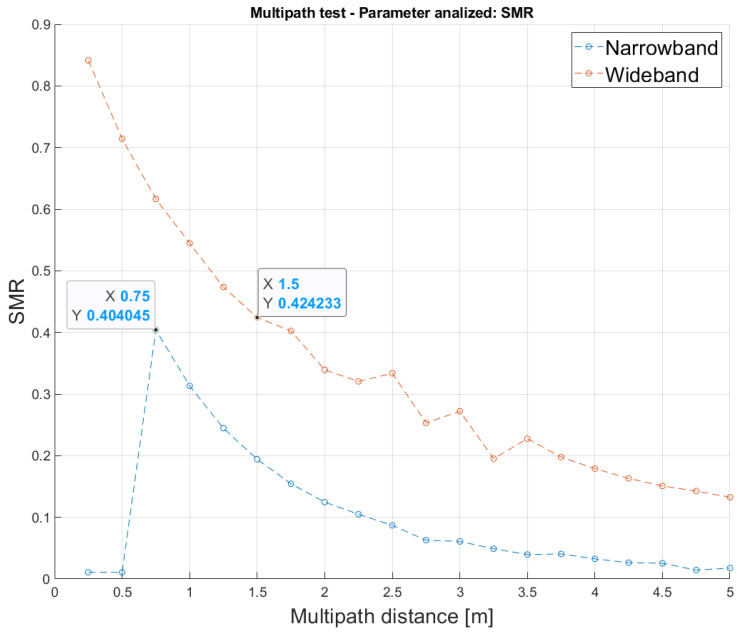
Average SMR according to the distance of the multipath replica, ranging from 0.25 m to 5 m in steps 0.25 m, with an SNR of 10 dB, for the narrowband and wideband modulations.

On the other hand, the multipath effect is one of the most significant issues in ultrasonic communications and localization systems. To study this effect, the multipath has been modeled as a replica of the original transmitted signal, affected by time delays and attenuations corresponding to the simulated paths. This replica has been placed at different multipath distances from the main reception, ranging from 0.25 m to 5 m in steps 0.25 m. Note that ten trials have been considered for every multipath distance. Figure 7 shows the average SMR achieved, according to the multipath distance variation. It is possible to observe that, in the narrowband approach, the multipath effect disappears below the distance (0.5 m) defined by the modulation symbol period, thus avoiding the detection of those multipaths close to the direct path. Furthermore, the rest of the paths also remain below the SMR threshold of 50% for proper operation, not disturbing the direct path. In contrast, the wideband modulation can detect other paths closer to the direct path, which may cause confusion between the direct path and the others, also providing much higher SMR values for the same multipath distance. This implies that the proposed narrowband approach is more resilient against the multipath effect.

**Figure 8:**
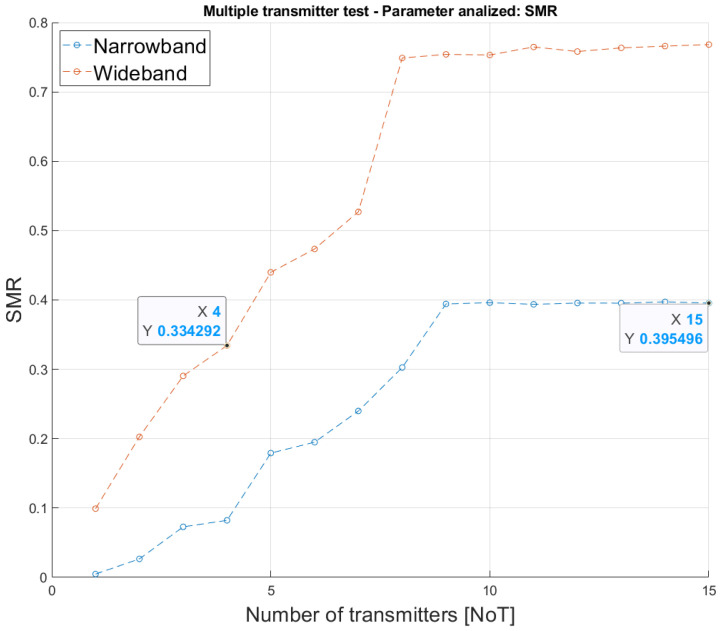
Average SMR for both the narrowband and the wideband modulations according to the number of simultaneous emitters, with an SNR of 10 dB.

A last aspect that was evaluated is the maximum number of simultaneous emitters supported by the proposal. Figure 8 shows the average SMR for the narrowband and wideband modulations as a function of the number of simultaneous transmitters existing in the environment, ranging from one to fifteen. Note that ten simulations were run for every number of transmitters. As shown in Figure 8, the maximum cross-correlation values keep the narrowband SMR below 40%, thus ensuring a suitable TDoA determination. In contrast, the wideband system reaches a limit with seven transmitters before cross-correlation values exceed 50% of the direct correlation peak.

### 4.2. Experimental Results

**Figure 9:**
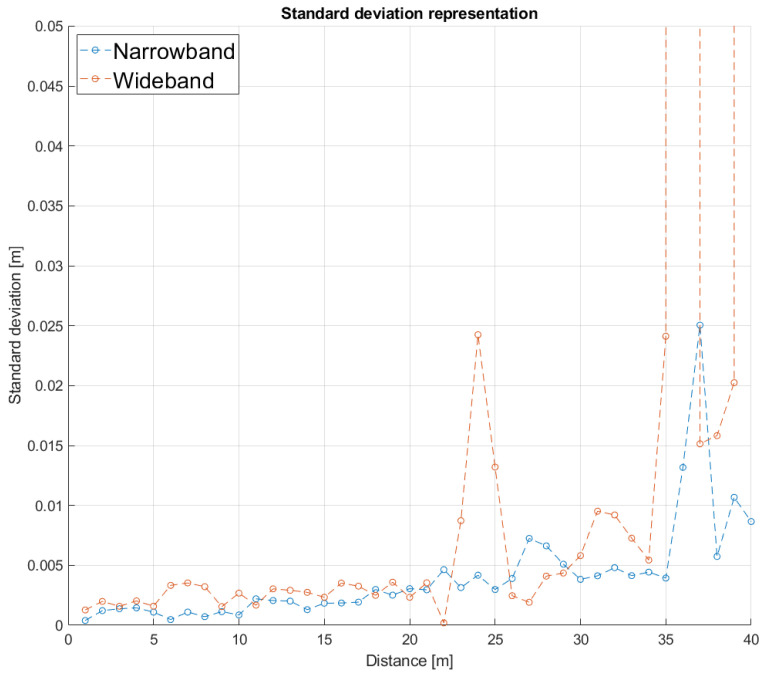
Standard deviation in meters for the determination of the correlation peaks, according to the distance between the emitter and the receiver in the experimental tests.

To validate the proposal’s performance, the variation in the SMR metric with respect to the distance between the emitter and the receiver has also been evaluated experimentally. In order to compare it with a similar test carried out previously in simulation with distances up to 30 m, the experimental setup was deployed in an indoor sports pavilion at the University of Alcalá. The indoor sport court is 40 m long and 20 m wide, so the transmitter was on one side, and the receiver was moved away in steps of 1 m. As before, the narrowband transmissions have a central frequency of 31 kHz, a bandwidth of 1 kHz, with a 255-bit Kasami code BPSK modulated. The wideband approach transmits the same 255-bit Kasami code modulated in BPSK with a carrier frequency of $f_{c}=41.67$ kHz. At every considered distance, an acquisition with an approximate duration of 20 s is carried out, which has allowed us to extract an average of 40 transmissions per second in the narrowband modulation and 100 transmissions in the wideband one. Figure 9 shows the standard deviation in meters for the time instant of the correlation peaks according to the distance between the emitter and the receiver, for both modulations. It is possible to observe that the wideband modulation presents a higher error in the determination of the correlation peak at shorter distances, compared to the narrowband one.

**Figure 10:**
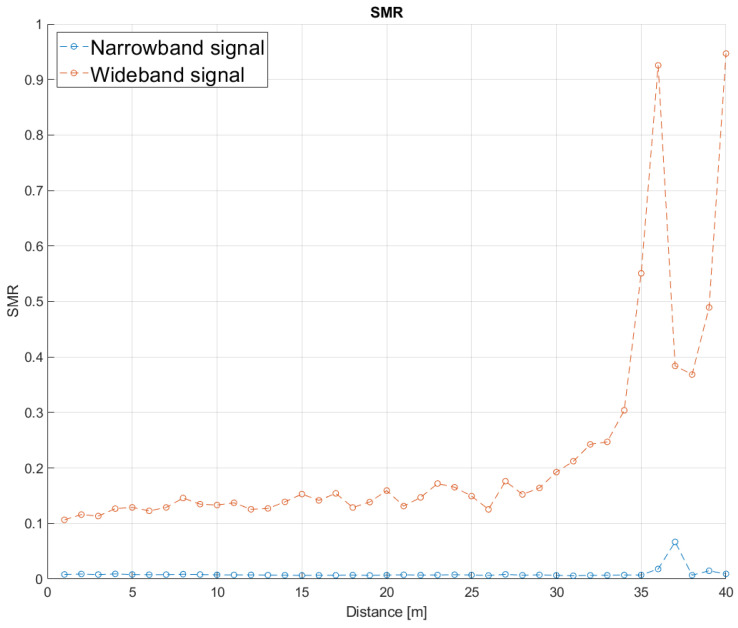
Averaged SMR according to the distance between the emitter and the receiver for both modulations, wideband and narrowband.

**Figure 11:**
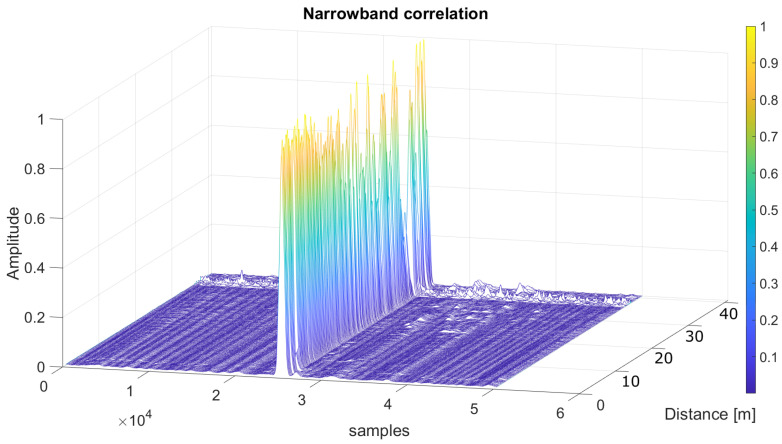
Normalized narrowband correlation functions depending on the distance between the transmitter and the receiver, ranging from 1 m to 40 m.

**Figure 12:**
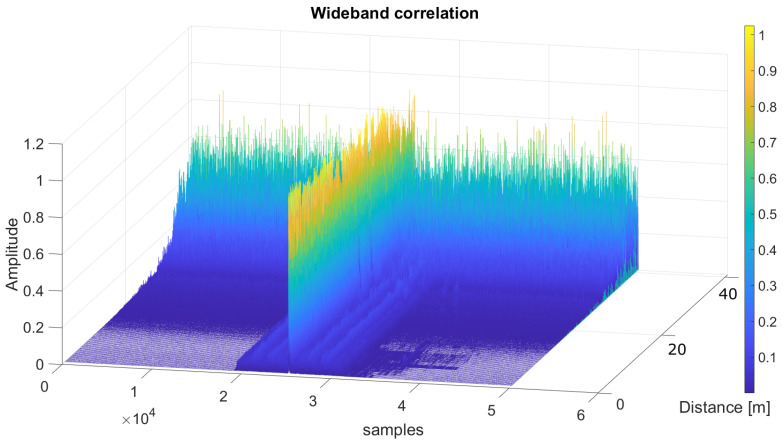
Normalized wideband correlation functions depending on the distance between the transmitter and the receiver, ranging from 1 m to 40 m.

In order to make a comparison with the results shown in the simulations, a representation of the averaged SMR for the same acquisitions has been plotted in Figure 10, according to the distance between the emitter and the receiver. As can be observed, the results obtained in the experimental tests are better than those from simulations, likely due to the constraining conditions fixed in the simulation in the digital twin to model the ultrasonic propagation. In this way, in simulations, the SMR for the narrowband scheme remained below 50% at distances up to 32.5 m, whereas in the experimental tests, the narrowband approach reached a distance of 40 m with an SMR below 50%. A similar improvement in the performance can be observed in the wideband modulation, with distances up to 34 m. Furthermore, Figure 11 shows the result of the normalized correlation functions for the narrowband modulation according to the distance between the emitter and the receiver, from 1 m to 40 m in steps of 1 m. It is worth noting that the correlation peaks appear clean and stable, with a certain degradation of 10% in the SMR for distances longer than 37 m. Finally, Figure 12 plots the same information as Figure 11, but for the wideband modulation, where two different aspects can be observed. The first one is the background noise generated by the correlation itself, which provides the best SMR results close to 0.1 even at the shortest distances, and the second one is how the noise significantly increases as the distance does. Note that it is difficult to identify the correlation peak for distances longer than 30 m.

Finally, it is worth noting that the proposed narrowband modulation may be applied to other application domains, such as ultrasonic imaging [48], where similar encoding and modulation schemes to those involved in positioning are often implemented, thus providing them with the same advantages detailed in this work. In a similar way, although the proof-of-concept validation presented here focuses on a single emitter–receiver configuration, the proposed method can be naturally extended to multi-channel MIMO implementations by distributing the coded subcarriers across different transmission paths. This provides a feasible pathway to combine the robustness of narrowband operation with the spatial diversity and scalability of MIMO ultrasonic systems.

## 5. Conclusions

A novel modulation technique has been proposed for ultrasonic sensory systems, such as those used in local positioning. The proposal consists of a multi-carrier architecture that uses spread spectrum encoding techniques and matched filtering. The proposed architecture allows the use of a single channel that can be adapted to any transducer frequency response, as well as the creation of multiple subchannels to operate with FDMA, offering high flexibility and improved performance. Furthermore, a non-coherent phase correlation method has been developed and implemented to enhance the correlation function, focusing on the detection of the subchannel’s energy arrival, which provides a more robust TDoA estimation against spectral noise and other significant effects in ultrasonic systems, such as multipath.

The proposed modulation scheme has been validated by evaluating the SMR ratio, which indicates how easily the correlation peaks can be detected. The technique has demonstrated superior performance and a stronger main correlation peak in different scenarios compared to conventional modulation schemes, such as wideband BPSK-based approaches. The simulated and experimental results show improved performance at longer distances and under lower SNR conditions. Future work will further assess its performance under typical adverse ultrasonic conditions, such as Doppler effects and multipath propagation. Furthermore, experimental scenarios with multiple simultaneous transmitters will also be considered, in order to test how the proposed scheme behaves under more demanding conditions with possible crosstalk interference from different emitters.

## Data Availability

Data are contained within the article.
